# Different Radial Modification Profiles Observed on APPJ-Treated Polypropylene Surfaces according to the Distance between Plasma Outlet and Target

**DOI:** 10.3390/polym14214524

**Published:** 2022-10-26

**Authors:** Fellype do Nascimento, Bruno Silva Leal, Antje Quade, Konstantin Georgiev Kostov

**Affiliations:** 1 Faculty of Engineering in Guaratinguetá, São Paulo State University—UNESP, Guaratinguetá 12516-410, Brazil; 2 Leibniz Institute for Plasma Science and Technology—INP, 17489 Greifswald, Germany

**Keywords:** DBD plasma, plasma jets, transferred plasma, plasma treatment, polymer treatment

## Abstract

The plasma jet transfer technique relies on a conductive wire at floating potential, which, upon entering in contact with a primary discharge, is capable of igniting a small plasma plume at the distal end of a long flexible plastic tube. In this work, two different long tube configurations were employed for the surface modification of polypropylene (PP) samples using argon as the working gas. One of the jet configurations has a thin copper (Cu) wire, which was installed inside the long tube. In the other configuration, the floating electrode is a metallic mesh placed between two plastic tubes in a coaxial arrangement. In the first case, the tip of the Cu wire is in direct contact with the working gas at the plasma outlet, whereas, in the second, the inner plastic tube provides an additional dielectric barrier that prevents the conductor from being in contact with the gas. Water contact angle (WCA) measurements on treated PP samples revealed that different surface modification radial profiles are formed when the distance (*d*) between the plasma outlet and target is changed. Moreover, it was found that the highest WCA reduction does not always occur at the point where the plasma impinges the surface of the material, especially when the *d* value is small. Through X-ray photoelectron spectroscopy (XPS) analysis, it was confirmed that the WCA values are directly linked to the oxygen-functional groups formed on the PP surfaces after the plasma treatment. An analysis of the WCA measurements along the surface, as well as their temporal evolution, together with the XPS data, suggest that, when the treatment is performed at small *d* values, the plasma jet removes some functional groups at the point where the plasma hits the surface, thus leading to peculiar WCA profiles.

## 1. Introduction

The surface modification of materials through the use of cold atmospheric pressure plasmas (CAPPs) has been a subject of intense research and development for both academic and technological purposes. This enhanced interest is caused by the fact that CAPPs are able to perform both chemical and physical action on the treated surfaces by creating/removing functional groups and changing the surface roughness [1,2,3,4]. For a large quantity of materials, surface modifications are required or desired to facilitate the occurrence of other processes, such as adhesion, or to enhance another surface property [5,6,7,8,9,10].

The device configurations that can be used to produce CAPPs for material treatment are quite diverse [5,11,12,13,14]. A few examples of systems that can operate in open environments are dielectric barrier discharge (DBD), discharge inside a funnel and atmospheric pressure plasma jets (APPJs) [12,15,16,17]. Planar DBD devices can be assembled in ways appropriate to treat materials with large area surfaces [18]. On the other hand, APPJs are able to provide localized surface treatment due to the small plasma plume dimensions of the typical plasma jets [19,20,21]. APPJs produced at the end of long and flexible tubes are able to treat complex surfaces as well as the ones inside cavities [22,23]. In addition, since the device is flexible, it can be mounted onto a scanning tool and adapted to treat large area surfaces.

APPJs can be produced by direct plasma extraction from the discharge in a dielectric enclosure or through the jet transfer technique (as a secondary plasma jet generated from a primary one) [13,22,24,25]. The advantage in the first case is that the voltage values required to ignite the discharge are, in general, lower than in the second. However, the jet transfer technique has as an advantage in that the high-voltage electrode is far from the plasma application spot. When the jet transfer technique is employed, the voltage that ignites the remote plasma jet is the one on the floating metallic conductor, which is lower than the applied voltage [24,25]. When APPJs are produced using the jet transfer technique, most of the species in the primary discharge are mainly the ones from the working gas atoms (in both excited and ionized states). However, some molecules from the air eventually enter the reactor chamber and sum up to others that originate along the pipes connecting the main gas bottle to the plasma device, becoming chemically active due to the ignition of the primary discharge. Nonetheless, the largest amount of active species is produced together with the secondary discharge, that is, the plasma jet, due to the interaction with the surrounding air.

When comparing the surface modification induced by punctual plasma sources, such as the plasma jets, to DBD systems that are able to treat large areas at once, the latter can achieve a better treatment homogeneity than the first. However the typical discharge gap in DBD systems is of the order of a few millimeters, thus limiting their application to thin flat samples. On the other hand, besides their small modification range, APPJs have a higher energy density per unit area; therefore, they perform a much faster treatment, and the surface modifications tend to be more intense [26]. Thus, the challenge of employing APPJs for the treatment of large objects and/or irregular objects is carrying this out in a homogeneous manner. Some works reported success in achieving uniformity in surface treatment by employing multiple plasma jets operating in parallel [27,28]. However, the individual plasma jets tend to interact with each other, making the operation of a plasma jets array a quite complex task.

A commonly used way to evaluate the interaction between plasmas and materials is to check if there were changes in the surface wettability, which is usually assessed by water contact angle (WCA) measurements, comparing the WCA values before and after plasma exposure [29]. Most of the works on polymer surface treatment by APPJs report similar modification profiles, obtained through WCA measurements, that resemble an inverted Gaussian profile [5,30,31]. A work by Naramisa et al. presents a slightly different WCA profile, with a deeper WCA reduction close to the spot where the plasma impinges the target [32]. A work by Shao et al. reported that the WCA reduction as a function of the distance *d* between the plasma outlet and the target surface does not present a monotonic behavior as a function of *d* when polymethyl–methacrylate samples are exposed to plasma treatment [33].

In this work, we report on the surface modification of polypropylene (PP) using APPJs. The treatments were performed using APPJs produced at the tip of long and flexible plastic tubes. Two different device configurations were employed in this study. Both configurations exploit the jet transfer technique, but their design concepts differ significantly. WCA measurements revealed that plasma treatment enhances the surface wettability and that the WCA radial profile along the surfaces changes significantly according to the distance between the plasma outlet and the target. X-ray photoelectron spectroscopy (XPS) analysis confirmed that the WCA profiles are related to the insertion of oxygen-functional groups on the polymer surface.

## 2. Materials and Methods

**Figure 1:**
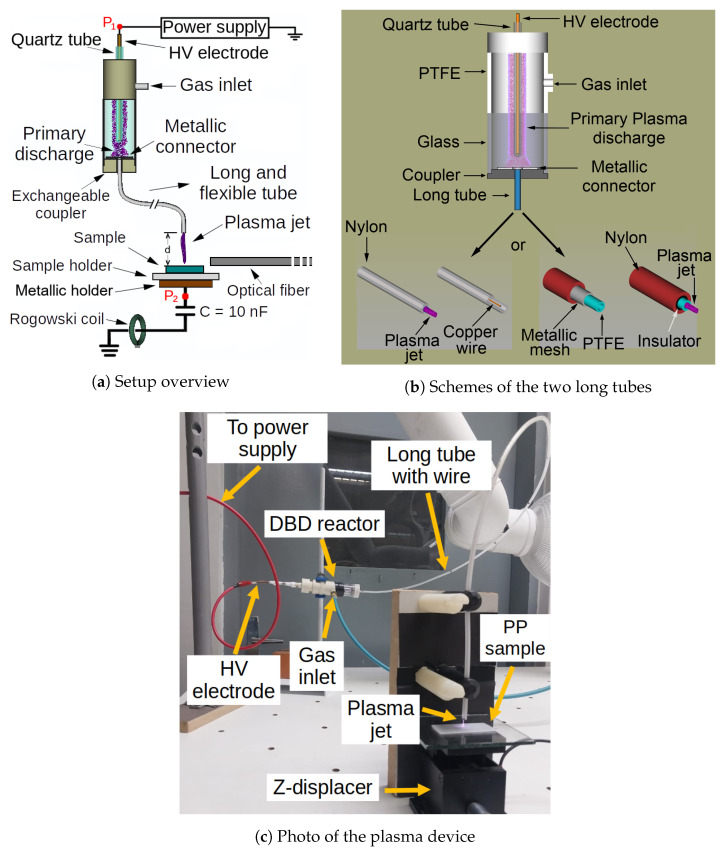
(**a**) Overview of the experimental setup. (**b**) Details of the two long tube configurations. (**c**) Photograph of the plasma device using the wire configuration, with the APPJ being applied on a PP sample.

Figure 1a shows a schematic of the experimental setup used in this work. A detailed view of the two long tube configurations is depicted in Figure 1b. The main plasma source presented in Figure 1 is a dielectric barrier discharge (DBD) reactor, composed of a metal pin electrode, whose diameter is 1.8 mm, encapsulated into a closed-end quartz tube with outer diameter of 6.0 mm and wall thickness equal to 2.0 mm, which, in turn, is placed inside a dielectric chamber with inner diameter of 24 mm. A 1-meter long flexible plastic tube is connected to the reactor exit. In this work, the long tube employed to produce APPJ was assembled in two different ways. In the first one, a 0.5 mm thin copper wire is inserted inside a plastic tube, whose material is Nylon 6, with outer diameter (OD) equal to 4 mm and inner diameter (ID) equal to 2 mm. In the second configuration, the long tube is formed by a metallic mesh, with 90% closed area, over a polytetrafluoroethylene (PTFE) tube with OD = 3 mm and ID = 2 mm, and both are placed inside a Nylon 6 tube with OD = 6 mm and ID = 4 mm. Both the wire and the mesh are fixed to a metallic connector that goes inside the reactor and acts as a floating electrode. Besides that, each set formed by the long tube and metallic connector is attached to its own exchangeable coupler, which is made of polyoxymethylene (POM). Furthermore, both copper wire and metallic mesh ends 2 mm before the plasma outlet. In addition, in the last configuration, there is an electrical insulation at the end of the mesh in order to avoid discharges coming directly from it.

In order to produce the plasma jets, the working gas, argon (Ar) 99.99% pure, is fed into the chamber and flows out through the long tube. When a high voltage (HV) signal is applied to the pin electrode, a primary discharge is ignited inside the reactor chamber. Once this primary DBD discharge is on, it polarizes the metallic connector that is connected to the floating wire or mesh inside the long tube. Eventually, the electric field induced on the floating electrode tip is sufficient to generate a small (secondary) plasma jet at the distal end of the plastic tube. The power supply employed to generate plasma jets in this work was composed of a commercial AC generator from GBS Elektronik GmbH (model Minipuls4) that works together with an arbitrary function generator from RIGOL (model DG1012) and a DC voltage generator. The DC supply was used to power the AC generator while the function generator was responsible for its voltage modulation. In the experiments performed in this work, the power supply was set up to deliver (340 ± 17) µJ in each voltage cycle. The voltage modulation was performed by producing a sequence with 15 rectangular voltage pulses at a frequency of 27.0 kHz followed by a voltage-off time interval. This process was repeated every 1.7 ms. Thus, the resulting HV waveform consisted of repetitive groups of sinusoidal waveforms (bursts), whose oscillation frequency ($f_{osc}$) was 27.0 kHz, followed by a voltage off interval. The entire HV signal repeated in a period τ = 1.7 ms. The amplitude modulation of the applied voltage signal (burst mode) helped to reduce the heating induced by an AC voltage and allowed for a better control on the mean discharge power.

The applied voltage was measured using a 1000:1 voltage probe (Tektronix, model P6015A) and its waveform was recorded on a 200 MHz oscilloscope (Tektronix, model 2024B). The signal of the current that passes through the system was acquired by a Rogowski coil from Pearson™ (model 4100), and it was used to calculate the effective discharge current ($i_{RMS}$).

In order to obtain the average discharge power ($P_{dis}$) dissipated along the entire device, simultaneous measurements of the voltage (V(t)) applied on the powered electrode, at node P${}_{1}$ in Figure 1a, and the voltage across a serial capacitor ($V_{C}\left(t\right)$, C = 10 nF), at node P${}_{2}$ in Figure 1a, were carried out. The charge variation on C ($q\left(t\right)=V_{C}\left(t\right)\times C$) during all voltage oscillations in a burst was thus recorded. Then, the $P_{dis}$ value was calculated by summing the area of the q−V Lissajous figures formed between the voltage and charge signals, divided by the burst period, which is [34,35,36]:

(1) $$P_{dis}=\frac{1}{\tau }\oint qdV$$

Since both *V* and *q* are time-dependent variables, Green’s theorem can be applied to (1) in order to rewrite the integration over a closed curve as an integration over time for parametrized quantities [37]. By carrying that out, we obtain:

(2) $$\oint qdV=\frac{1}{2}\int_{t_{1}}^{t_{2}}\left[V\left(t\right)q^{\prime }\left(t\right)-V^{\prime }\left(t\right)q\left(t\right)\right]dt$$

where $V^{\prime }\left(t\right)=dV\left(t\right)/dt$ and $q^{\prime }\left(t\right)=q\left(t\right)/dt$. Thus, $P_{dis}$ can be expressed as:

(3) $$P_{dis}=\frac{1}{2\tau }\int_{t_{1}}^{t_{2}}\left[V\left(t\right)q^{\prime }\left(t\right)-V^{\prime }\left(t\right)q\left(t\right)\right]dt$$

**Figure 2:**
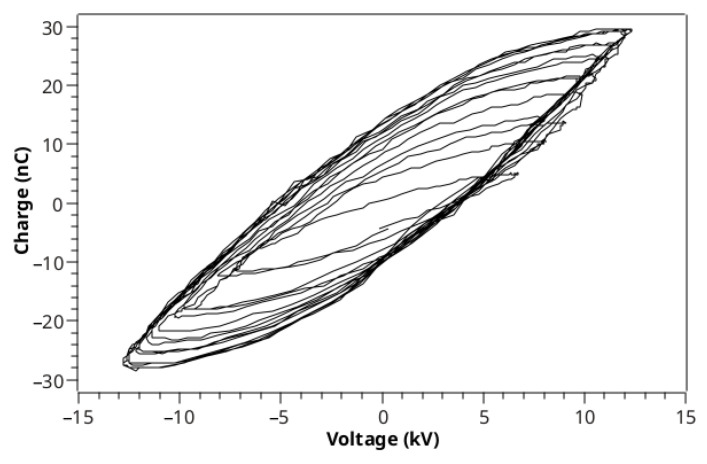
Example of a q−V plot with 15 oscillation cycles.

Equation (3) has the advantage of being able to be used to calculate the area of the Lissajous figure without the need to plot a q−V curve because it is carried out numerically using the V(t) and q(t) waveforms. This is especially useful when dealing with q−V plots containing multiple cycles, such as the one shown in Figure 2.

Broad-band optical emission spectroscopy (OES) in the wavelength range of 200 nm to 750 nm was performed using a spectrometer from Avantes (model AvaSpec-ULS2048X64T) with a spectral resolution (FWHM) of 0.76 nm. An optical fiber was positioned parallel to the surface target to gather the light emitted by the plasma jet. The distance between the center of the plasma column and the fiber optic light input was 5 mm.

Rotational and vibrational temperature values ($T_{rot}$ and $T_{vib}$, respectively) of $N_{2}$ molecules were assessed using spectroscopic measurements. In order to obtain those $T_{rot}$ and $T_{vib}$ values, we used spectroscopic emissions from the $N_{2}$ second positive system, $C{}^{3}\Pi_{u},\nu^{\prime }\to B{}^{3}\Pi_{g},\nu^{\prime \prime }$, with Δν = $\nu^{\prime }-\nu^{\prime \prime }$ = −2, in the wavelength range of 362 nm to 382 nm [38,39,40,41]. Simulations of emission spectra were carried out using an appropriate software (massiveOES, for instance) [42,43]. In this manner, comparisons between the observed and simulated spectra were made, and temperature values were picked by those simulations that provided the best-fitting between simulated curves and experimental spectra. It is known that spectroscopic measurements performed with low-resolution spectrometers, such as the Avantes one, are not sufficient to fully resolve the rotational levels of the $N_{2}$ molecules, which is a requirement for obtaining accurate values for the $T_{rot}$ parameter. However, the shape and broadening of the $N_{2}$ vibrational bands and the variation in the $T_{rot}$ values are directly related, being that the higher the $T_{rot}$, the larger the broadening and also the higher the intensity of the light emissions coming from the rotational lines inside the vibrational bands. Both effects change the shape of the vibrational bands in the segment that degrades to violet, making them become higher and wider, which allows for the estimation of $T_{rot}$ values by using low-resolution spectrometers. In this way, even if they are not so accurate, the $T_{rot}$ values obtained with low-resolution spectrometers can be good enough to reveal the trend of that parameter in the experiment carried out in this work.

The APPJs produced with both configurations—long tube with wire and mesh—were applied on polypropylene (PP) samples in order to evaluate the surface modification generated by each treatment. The high-density (0.95 g/cm${}^{3}$) PP material used in this work is commercially available. All of the samples were obtained from a 1 mm-thick sheet and were cut in approximately the same dimensions (35 mm × 25 mm). Before exposure to plasma treatment, the samples were cleaned with an ultrasound cleaner in a beaker with cleaning substances. First, the samples were washed with deionized water and detergent for 10 min. After that, they were rinsed with isopropanol (99.9% purity) for 20 min to remove organic compounds from the surfaces. The samples were then washed with deionized water for 20 min more to remove any remaining contaminants. Finally, they were left to dry in a controlled environment at room temperature. In order to perform the surface treatment, the PP samples were placed on a sample holder made of glass (dimensions: 10 cm × 10 cm × 5 mm-thick) and, after that, exposed to the plasma jet. The degree of PP surface modification was analyzed by measurements of water contact angle (WCA). Additionally, the effects of APPJ treatments on the surface of selected PP samples were analyzed through X-ray photoelectron spectroscopy (XPS). The XPS measurements were performed with equipment from Kratos (model Axis Supra), whose energy resolution for polymers is better than 1 eV and whose local resolution is better than 15 µm.

## 3. Results and Discussion

### 3.1. Electrical Parameters for the Different Configurations

**Figure 3:**
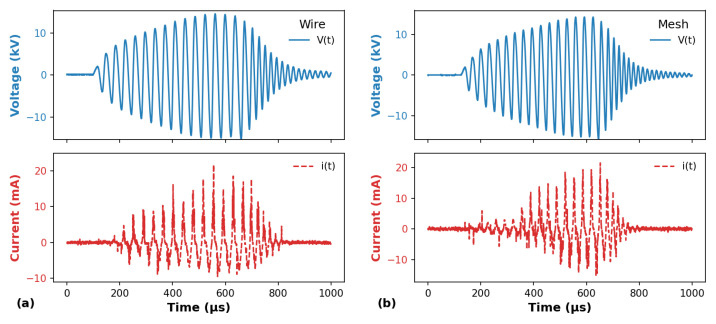
Typical waveforms of applied voltage (V(t)) and discharge current (i(t)) using (**a**) wire and (**b**) mesh configurations. The gas flow rate was 2.0 L/min in both cases.

Figure 3 shows the typical waveforms of the applied voltage and the corresponding current signal for the plasma jets generated using (a) the wire configuration and (b) the mesh one. In both cases, Ar at a flow rate of 2.0 L/min was employed as the working gas and the plasma jet impinged the glass plate. By comparing the voltage waveforms presented in Figure 3a,b, it can be verified that the use of different tube configurations does not lead to significant changes in the voltage waveforms, either in the amplitude or in the behavior. Regarding the electrical current waveforms, the most notable difference between the two cases is that the current signal in the mesh configuration presents a shorter duration, with an apparent delay in the current growth. Another point that can be noticed is that the current peak values for the signal positive phase have about the same magnitude. However, i(t) presents slightly higher negative values when the mesh mounting is employed.

The discharge power ($P_{dis}$) and the electrical current, presented in this work as the root mean square value of the current waveform ($i_{RMS}$), are the most important electrical parameters in APPJs. Usually, for biomedical applications, it is desirable to have APPJs with sufficient electrical power to generate plenty of reactive species but a low electrical current. This combination is especially required for in vivo applications. However, plasma jets with low $P_{dis}$ values are also useful when soft materials are subjected to APPJ treatment.

**Figure 4:**
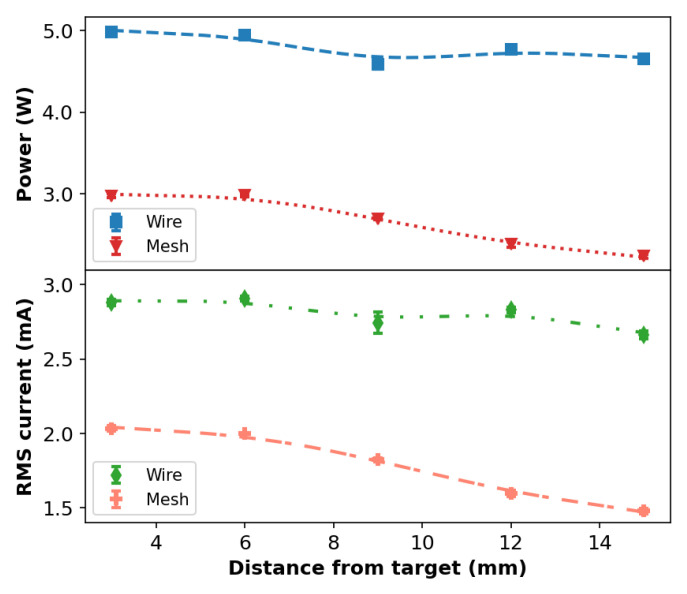
Discharge power and effective current as a function of the distance between plasma outlet and target obtained with a gas flow rate of 2.0 L/min.

Figure 4 shows the behavior of $P_{dis}$ and $i_{RMS}$ as a function of the distance (*d*) between the plasma outlet and the target using Ar at a flow rate of 2.0 L/min. From Figure 4, it can be seen that, for the wire or mesh configurations, both $P_{dis}$ and $i_{RMS}$ curves present very similar trends as a function of *d*; however, they have different amplitudes. The average $P_{dis}$ values are ~40% higher in the wire configuration when compared to the mesh one. In general, the $i_{RMS}$ values are ~35% higher when the wire is employed.

The explanation for the lower values of $P_{dis}$ and $i_{RMS}$ obtained for the mesh configuration is quite simple: when compared to the tube with a wire inside, the mesh configuration provides an additional dielectric barrier between the mesh electrode and the gas flow. Thus, in this way, the electrical safety of the equipment is improved, making it, for example, suitable for manual handling and in vivo applications. Moreover, the lower discharge intensity and duration obtained with the mesh configuration also contributes to the lower $P_{dis}$ values in that case.

### 3.2. Spectroscopic Emissions and Thermal Parameters

**Figure 5:**
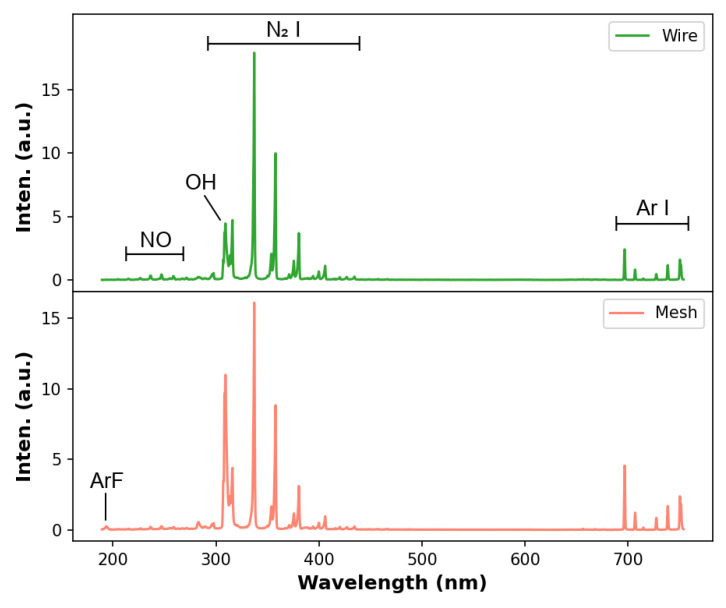
Overview of the emission spectra of the plasma jets obtained using wire (**top**) and mesh (**bottom**) configurations. The distance between plasma outlet and target was 6 mm and the gas flow rate was 2.0 L/min.

Figure 5 shows an overview of the emission spectra of the Ar plasma jets obtained using the wire and mesh configurations. Both spectra shown in Figure 5 exhibit band emissions from NO, in the 200 nm to 270 nm wavelength range, and OH at 288 nm, 296 nm and 308 nm, with the last two bands overlapped by $N_{2}$ emissions, and from $N_{2}$ molecules in the 298–450 nm range. Line emissions from argon atoms are also detected in each case, in the wavelength range of ~690 nm to 750 nm, and are indicated in Figure 5. Of course, other Ar line emission are present in the plasma jet, mainly above 750 nm, but they were not detected due to the limitation in the wavelength range of the spectrometer used in this work. Regarding the configurations used, there are no significant differences in the emitting species when the plasma jet is produced using wire or mesh. The only noticeable difference, detected in the spectra acquired using the mesh configuration, is a low-intensity ArF molecular emission close to 193 nm. That ArF emission is probably due to the interaction between the Ar plasma jet with the wall of the PTFE tube, which was used in the long tube mesh assembling. Such an emission was already reported in another study that also employed Ar as the working gas, together with a PTFE tube [19]. From Figure 5, it can also be noticed that the intensity emissions from OH and $N_{2}$ are relatively higher than the intensity emissions from Ar atoms. The intensity ratio between atomic and molecular emissions in APPJs can change according to the axial position of the plasma column, as was verified by Onyshchenko et al.; for example, [44]. Considering that, the atomic line emissions tend to be more intense than the molecular ones when the OES measurements are performed close to the plasma outlet, and the opposite tends to occur when OES is carried out far from the plasma outlet or close to a target surface; in this work, we chose to carry out the latter. Such a reduction in the relative intensity between atomic and molecular emissions is mainly due to the energy exchanged from the carrier gas to the molecules in the ambient air.

Among the thermal parameters of the APPJs produced using the configurations under study, we evaluated only the rotational and vibrational temperatures ($T_{rot}$ and $T_{vib}$, respectively). These two thermal parameters can be considered the most important ones for APPJs, since $T_{rot}$ has a close relationship with the gas temperature ($T_{gas}$), with $T_{rot}\approx T_{gas}$ in most cases, and $T_{vib}$ is related to the rate of chemical reactions [38,45,46,47]. It is important to mention that, when Ar is the working gas, the $T_{rot}$ values measured using band emissions from $N_{2}$ (C→B) are not the same as $T_{gas}$, as was extensively discussed in a review paper by Bruggeman et al. [38]. The main reason for that is because, when Ar is the working gas, the population and excitation of rotational levels of the $N_{2}$(C) vibrational band are not dominated by electron impact collisions, but by collisions with Ar atoms and $N_{2}$ molecules in metastable states, which can produce $N_{2}$(C) excited states with specific rotational distributions. It is also important to mention that the $T_{rot}$ values obtained using OES with Ar as the working gas are usually higher than the $T_{gas}$ ones, even when $T_{rot}$ is measured using emissions from OH radicals, like what was reported recently in [47].

**Figure 6:**
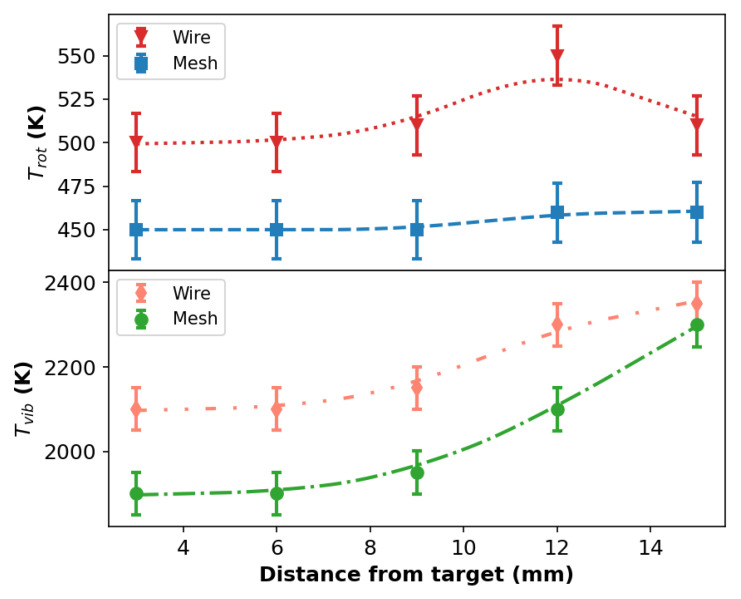
Rotational and vibrational temperatures as a function of the distance from target for both wire and mesh configurations with the APPJ impinging a glass target. The Ar flow rate was 2.0 L/min.

Figure 6 shows the variation in $T_{rot}$ and $T_{vib}$ as a function of the distance between the plasma outlet and the target calculated for both wire and mesh configurations. From Figure 6. it can be seen that the $T_{rot}$ values for the wire configuration are higher than those obtained with the mesh. In any case, such values are in agreement with ones reported in the literature for Ar plasma jets impinging on a dielectric surface [41,47,48]. Despite the difference observed in the values of $T_{rot}$ for the different configurations, both curves present similar trends as a function of *d*. The values of $T_{vib}$ also present a similar tendency for both configurations, increasing as *d* is incremented. The $T_{vib}$ values obtained with the wire mounting are slightly higher at small *d* values, but, at larger *d*, the $T_{vib}$ values are almost the same for both configurations.

### 3.3. Modification Profile of PP Samples

The effects of plasma treatment on the surfaces of PP samples using both wire and mesh configurations were evaluated through measurements of the water contact angle (WCA) and X-ray photoelectron spectroscopy (XPS). In all cases, the treated and non-treated samples were compared. In addition, the spatial distributions of the plasma treatments were analyzed. In samples that were submitted to APPJ treatment, the plasma jet was directed to a position close to their center. In all cases studied in this section, the working gas employed in the plasma treatments was Ar at a flow rate of 2.0 L/min, and the duration of the treatments was always 50 s, except when a different duration is indicated.

#### 3.3.1. WCA Analysis

**Figure 7:**
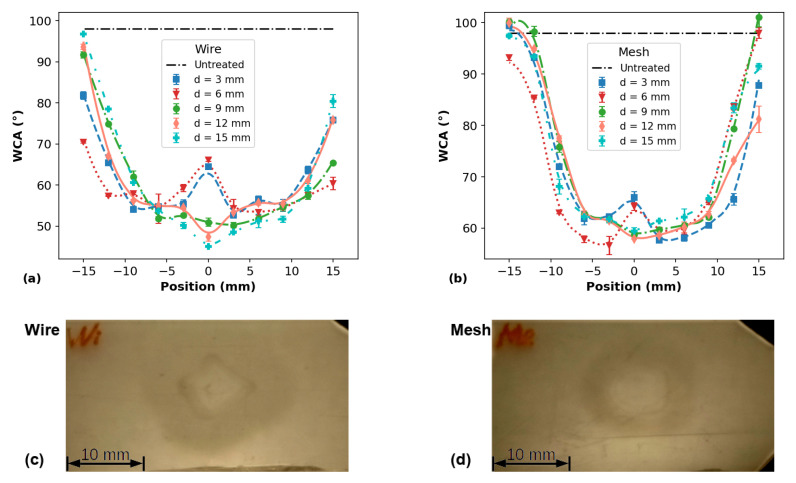
WCA profiles on PP surfaces measured for different distances between plasma outlet and target for (**a**) wire and (**b**) mesh configurations and corresponding photos of PP samples treated with (**c**) wire and (**d**) mesh when d = 6.0 mm.

Figure 7 shows the WCA profiles of PP surfaces, for different distances between the plasma outlet and target, measured soon after the APPJ treatment using (a) the wire configuration and (b) the mesh one. The corresponding photos of two samples treated with the wire and mesh jet configuration (for *d* = 6 mm) are also shown in Figure 7c,d, respectively. It is possible to see that, in both cases, the plasma jet left a visual “fingerprint” on the PP surface. Its shape resembles a donut whose size more or less correlates to the dimension of the visual plasma spreading on the sample surface (~12 mm). Thus, one can make a guess that the plasma effect would be limited to this visually different area on the sample surface.

The WCA measurements were performed at different positions (*l*) along a line parallel to the larger sample dimension and passing through the point where the plasma jet was directed, that is, the geometrical center of the sample (*l* = 0 mm in the curves). The WCA value of the untreated PP samples is ~98°. From Figure 7a,b, it can be seen that both wire and mesh configurations are able to promote a reduction in the WCA of the PP surface. The WCA profiles also allow us to evaluate what the treatment efficacy of each plasma jet is, as well as the size of the area affected by the treatment. Using the plasma jet with the wire configuration caused an average reduction of 45° in the WCA for a range of ~20 mm (using the FWHM as a parameter), whereas, when using the mesh configuration, the average WCA reduction was 40° over a slightly smaller range. In both cases, the surface modification effect extends over an area larger than the plasma jet fingerprints in Figure 7c,d. The WCA reduction at the positions far from the center of the samples is probably caused by the PP surface interaction with long-lived reactive species produced by the plasma jet, which are carried by the gas flow. In addition, another possibility for marginal WCA reduction can be an accumulation of low-molecular-weight oxidized fragments (LMWOFs) that formed at the central region of the PP surface and were dragged to the borders by the Ar flow [49].

When applying the two different plasma jet arrangements for PP treatment, the values of the discharge power and the $i_{RMS}$ were also different: $P_{dis}$ = (3.1 ± 0.2) W and $i_{RMS}$ = (1.83 ± 0.09) mA for the wire configuration and $P_{dis}$ = (2.7 ± 0.3) W and $i_{RMS}$ = (0.92 ± 0.05) mA for the plasma jet with mesh, respectively. Consequently, the rotational and vibrational temperature values obtained when the sample holder was a dielectric material were: $T_{rot}\approx 600$ K and $T_{vib}\approx 2300$ K for wire and $T_{rot}\approx 500$ K and $T_{vib}\approx 1900$ K for mesh. Therefore, taking into account that all of these parameters have higher values when the wire configuration is employed, we can infer that the higher reduction in the WCA values are due to a synergy among them.

In order to check for thermal effects of the APPJ on the PP samples, measurements of the temperature on the PP surface were carried out immediately before plasma exposure and also immediately after ten minutes of plasma treatment. For such purposes, we employed an infrared thermometer from Minipa (model MT-395). With this procedure, we observed that the temperature variation on the PP sample surface was lower than one Celsius degree. In addition, since the PP material has a low heat capacity, any possible local heating would not spread far from the spot in which the plasma jet impinges on the target, that is, the center of the sample. In addition, due to the spread of the plasma jet over the surface, the thermal energy per unity area decreases as the spreading radius increases, which enforces the fact that thermal effects would not reach the sample’s borders. Based on these information and also on the fact that the $T_{gas}$ is probably lower than the $T_{rot}$ we have measured, it is safe to say that the thermal effects play a minor role in the aforementioned results.

The WCA distributions shown in Figure 7a,b exhibit distant shapes for different treatment distances. For instance, for both long tube configurations at *d* equal to 3.0 and 6.0 mm, the longitudinal distributions of WCA profiles present unusual shapes, with the WCA values at the samples’ center being higher than in their neighborhood. In both wire and mesh cases, the WCA reduction at the center is ~40°, whereas, on the adjacent points, the WCA is ~45°, on average. The occurrence of these depressions in the WCA close to the center of the PP samples is likely due to the removal of some polar functional groups that were formed on the surfaces during the APPJ treatment. This is corroborated by the XPS analysis of the PP surface elemental composition after plasma treatment, which is presented in Section 3.3.2. However, when using the wire mounting, for higher *d* values (12.0 and 15.0 mm), the WCA reduction at the sample center was higher than those measured in the neighborhood. Similar behavior in the WCA distribution was already observed on plasma-treated polyethylene terephthalate (PET) by Narimisa et al. [32]. In that work, the authors used a pin-ring electrode configuration to produce a plasma jet using Ar as the working gas.

The small asymmetries in the WCA distributions shown in Figure 7 are probably related to a misalignment of the plasma jet impinging point with regard to the samples’ center. In addition, it is not always possible to ensure a normal incidence of the plasma plume on the sample surface. As a result, the reactive species distribution on the sample is not perfectly uniform, leading to slightly asymmetric WCA profiles.

**Figure 8:**
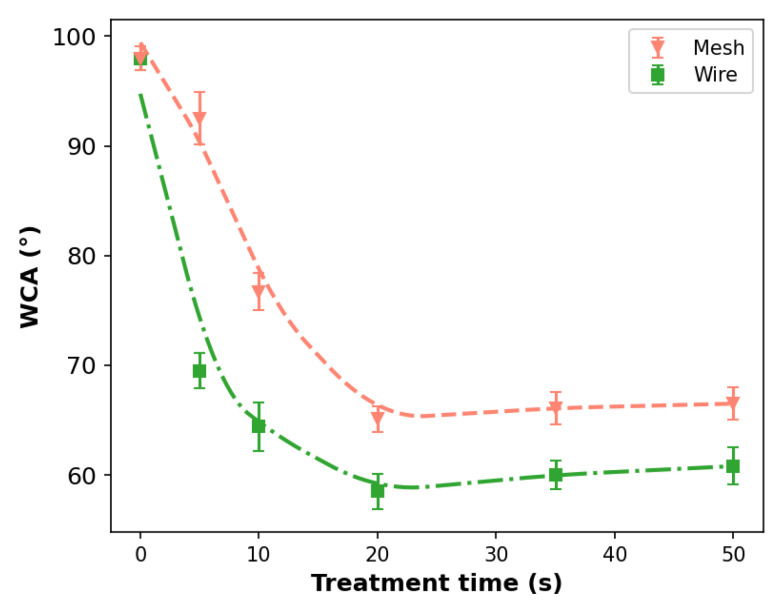
WCA measurements as a function of the treatment time. The distance between plasma outlet and target was 6 mm in this case.

The effects of the treatment time in the range of 0 s (non-treated case) to 50 s on the WCA in both PP were also analyzed, and they are shown in Figure 8. In these cases, the WCA measurements were performed only at the center of the samples. From Figure 8, it is clear that the plasma jet produced with the wire configuration is able to reduce the WCA values faster than the plasma jet obtained with the mesh. The former jet configuration is also more effective at reducing the WCA in the time period under consideration.

The behavior of the WCA values as a function of the treatment time are almost the same in both wire and mesh configurations, presenting a decrease for treatment times up to 20 s and achieving a saturation value after that. The temporal behaviors of WCA values for the PP samples treated using the wire configuration shown in Figure 8 together with the corresponding longitudinal WCA profiles shown in Figure 7 corroborate with the hypothesis that, during the APPJ treatment, simultaneously with the creation of polar groups on the PP surface, an etching process occurs, especially at the point where the APPJs impinge on the surface, leading to the removal of some functional groups and eventually achieving a steady-state.

**Figure 9:**
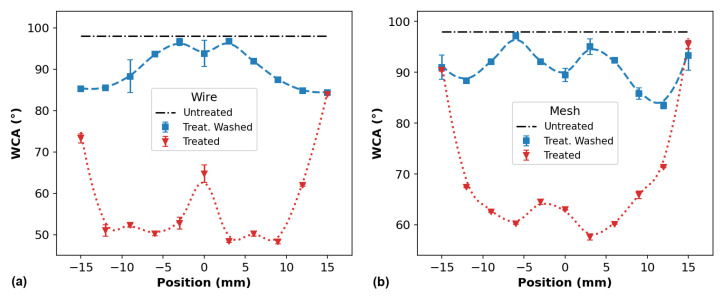
Comparison between WCA profiles of treated PP samples before and after washing for the APPJ treatment performed with (**a**) wire and (**b**) mesh mountings. The distance between plasma outlet and target was 6 mm.

Usually, polymer surfaces subjected to plasma treatment are prone to so-called hydrophobic recovery [50,51], i.e., with the time of storage, their WCA tends to increase. This process is caused by the gradual reduction of the polar groups drafted on the PP surface due to different processes, such as reorientation, evaporation, reaction with air molecules, etc. The plasma treatment of polymers is known to produce large amounts of short polymer fragments that are highly oxidized and tend to agglomerate into mount-like structures called low-molecular-weight oxidized material (LMWOM). These are loosely bound to the surface and can be removed by different processes, such as rinsing with a polar liquid [52]. In order to evaluate the stability of the WCA values on PP samples after plasma exposure, two other samples were treated, one for each long tube configuration, and new WCA measurements were carried out. The distance between the plasma outlet and target was kept at 6.0 mm in both cases. After that, the two samples were washed with deionized water in an ultrasound cleaner for 20 min. Through this process, the LMWOM is dissolved and the treated polymer samples mimic a long-term aging effect. Then, the WCA measurements were performed again at approximately the same positions as when they were performed before the washing. The WCA results before and after washing are shown in Figure 9.

An interesting point to be noted in Figure 9 is that the WCA recovery in both PP samples was more pronounced at their centers than at their borders. As stated before, when the plasma jets impinge the PP samples, their visible spread diameters are not longer than 12 mm. Therefore, taking into account all that information, one can speculate that the WCA reduction away from the spot where the plasma jets impinge the samples (at *l* = ±15 mm, for example) is not only caused by the agglomeration of LMWOM dragged by the gas flow because, otherwise, all of it should be removed by the washing process, and the WCA of the samples would recover to its original value.

#### 3.3.2. XPS Analysis of APPJ-Treated PP Samples

**Figure 10:**
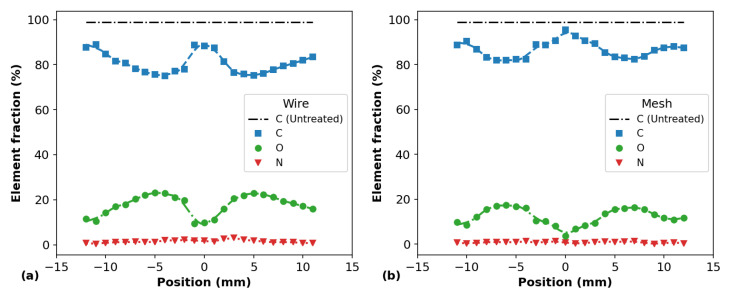
Fractions of the elements detected on the on the surface of PP samples for different positions through XPS measurements for (**a**) the wire configuration and (**b**) the mesh one.

**Figure 11:**
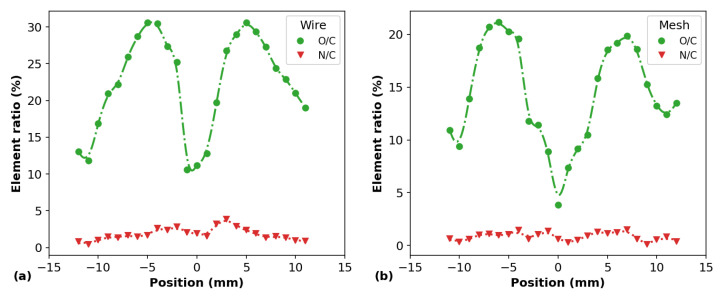
O/C and N/C element ratios obtained from XPS measurements on the surface of PP samples for different positions obtained for (**a**) the wire mounting and (**b**) the mesh one.

**Figure 12:**
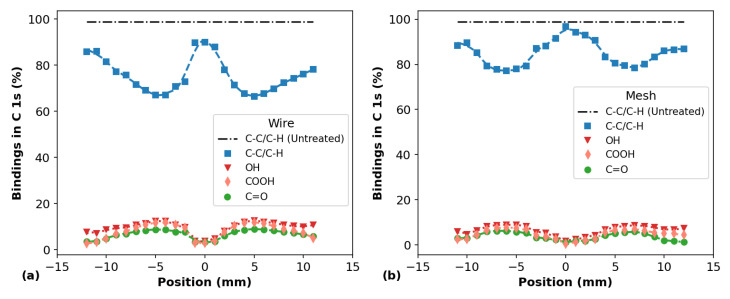
Bindings in C 1 s obtained from XPS measurements on the surface of PP samples for different positions using (**a**) the wire mounting and (**b**) the mesh one.

The surface elemental composition of the plasma-treated PP samples, for *d* = 6.0 mm, was investigated by XPS analysis. The obtained results are presented in Figure 10, Figure 11 and Figure 12. Figure 10a,b show the longitudinal distributions of the element fractions detected on the sample surfaces using the wire and mesh configurations, respectively. In both cases, reductions in the carbon (C) content on the surfaces were observed, which were accompanied by increments in the oxygen (O) content at the same positions. Small amounts of nitrogen (N) atoms were also detected on the samples after APPJ treatment. When the mesh configuration was employed, traces of fluorine (F) were also detected on the PP surface, probably detached from the PTFE tube due to the plasma–material interaction and driven to the target by the Ar gas flow. Importantly, the elemental distributions obtained by the XPS are in close agreement with the WCA distributions obtained for the PP samples treated at distance *d* = 6.0 mm. This can be better seen in Figure 11a,b, where the N/C and O/C element ratios are shown. Indeed, the O/C ratios for both wire and mesh APPJ treatments present a sharp depression of their values at the center of the samples, that is, in the positions where the plasma jets impinged the PP surfaces. These O/C curves make the reduction in the C fraction and the increment in the O fraction shown in Figure 10 more evident.

The N/C ratios shown in Figure 11a,b do not change in the same proportion as in the O/C ratios. Apparently, it seems that the N/C ratios of PP samples exhibit similar but much less expressive behavior. However, they clearly show that the insertion of N into the PP surfaces is higher when the wire configuration is used. In the N/C profile obtained for the wire configuration, a smooth depression in the curve can be noticed, which is not observed when the mesh is employed.

Finally, Figure 12 shows the bindings in C 1s obtained after the peaks deconvolution of the APPJ-treated samples using the components C-C/C-H, C-OH/R, C=O and COOH/R, whose binding energies are 285.0 eV, 286.7 eV, 287.8 eV and 289.2 ± 0.2 eV, respectively. The APPJ treatments performed with both wire and mesh configurations present reductions in the C-C/C-H components and increments in the C-OH/R, C=O and COOH/R ones as a consequence of the reduction in the C fraction and increase in the O fraction, respectively. It can be seen in Figure 12 that the longitudinal profiles of the C-C/C-H components and of the O-containing ones present similar shapes of the curves for the C and O element fractions (Figure 10), respectively. The linescan on the surfaces of the plasma-treated samples proves the insertion of O-containing functional groups (hydroxyls, carbonyls, carboxyls) across the line as the O-fraction is increased compared to the non-treated PP-material.

Concerning the differences in the amount of functional groups detected on the PP surfaces after APPJ treatment with different configurations, we can see that the wire mounting provides a higher reduction in the C-C/C-H components and a more efficient insertion of O-containing ones compared to the employment of the mesh mounting. This is consistent with the higher values of the discharge power and rms current, as well as temperatures, obtained for the wire arrangement.

By analyzing the set of results obtained through XPS measurements, we can safely state that the presence of O-containing functional groups and the reduction in C-C/C-H bonds on the PP surfaces are the main reasons behind the WCA reduction presented in Figure 7 and Figure 8. Moreover, by analyzing the WCA results together with the XPS ones, we can infer that an etching process takes place and that the O-containing functional groups inserted on the PP surfaces tend to be removed, especially at the samples’ center, where the plasma jet touches the polymer surface. The removal of such groups probably occurs at the same time as when they are inserted on the material under APPJ exposure. However, the etching process becomes dominant if the material is exposed to plasma treatment for a long time, leading to WCA saturation.

According to Arefi-Khonsari et al. [49], the PP material possesses a tertiary carbon bearing a methyl group with an isolated hydrogen, which makes it more sensitive to degradation, which leads to the formation of low-molecular-weight oxidized materials (LMWOMs). Thus, the patterns observed on the PP-treated surfaces, through photographs, WCA measurements and XPS analysis, can be due to the formation of LMWOMs at the positions where the APPJs impinge the sample surface, followed by a radial dragging of them. This statement is partially supported by the fact that the washing process performed on treated PP samples promoted a highly significant WCA recovery on the material surface in the region ranging from −10 mm to 10 mm from the samples center.

## 4. Conclusions

In this work, we report on the different modification profiles of APPJ-treated PP surfaces. The experiments were carried out using two different plastic tube configurations to generate plasma jets with different parameters. Both arrangements work using the jet transfer technique, which allows for the generation of a plasma plume on the distal end of a long plastic tube. One of them employs an assembly that uses a metallic mesh between two plastic tubes in a concentric arrangement. The plasma jet parameters obtained with this system, as well as the results obtained from the PP treatment with it, were compared to those from a device configuration that employs a long tube with a metal wire inside it. The comparison of the surface treatments of the PP polymer using both systems showed that the plasma jet in the wire configuration generates a plasma plume with the set of parameters, which gives better results in terms of the WCA reduction and the insertion of polar functional groups on the samples surface. In addition, the WCA and XPS measurements revealed that the APPJ treatment can not only insert O-containing functional groups on the polymer surfaces, but can also cause a removal of such groups, especially when the materials are under APPJ treatment for a long time.

Taking into account the modification profiles using both wire and mesh configurations, it is possible to conclude that the wire configuration was more effective in promoting surface modification on the PP samples exposed to APPJ treatment. However, if the treatment results are weighted with the $P_{dis}$ values and the discharge duration, we can say that the mesh configuration has the potential to produce almost the same results as the wire one, but with a lower discharge current, therefore having the advantage of a safer operation, which is essential, especially in in vivo applications.

One of the interesting findings in this work was the observation that the reduction in the WCA value at the spot where the plasma jet touches the sample can be smaller than the WCA measured in the vicinities when the sample is close to the plasma outlet. This is something that has not yet been reported in the literature. In addition, based on the results obtained comparing the WCA values measured on the PP samples exposed to APPJ treatment and washed afterwards, it was also found that the most stable surface modification occurs out of the region, where it is possible to visualize the spread of the plasma jets on the surfaces. This is a point that can stimulate further research for a better understanding of the surface hydrophilization process through APPJ exposure. Finally, combining data from WCA and XPS measurements, it was possible to find evidence that indicates that part of the functional groups created on the PP surface in the initial treatment interval can be removed if the APPJ treatment continues for a longer time.

In future work, we intend to investigate whether it is possible to avoid or reduce the etching process observed for treatment times higher than 20 s. In addition, an investigation regarding possible changes in the surface morphology of the PP samples exposed to APPJ treatment at different gas flow rates and distances between the plasma outlet and target will be carried out.

## Data Availability

Data are contained in this manuscript. Raw data are available from the authors under reasonable request.
